# P-296. Telehealth as a Modality to Improve the Uptake of PrEP Services in Black and Latino MSM "ePrEP"

**DOI:** 10.1093/ofid/ofaf695.517

**Published:** 2026-01-11

**Authors:** Cynthia Firnhaber, Aaloke Mody, Mitch Scoggins, Billie J Thomas, Benjamin Crouse, Sarah Chandler, Natalie Kallhoff, Hailey Keeser, Alex Camp, Kesley Bohr, Brendan DeMarco, Amanda Partee, Leslie Cockerham

**Affiliations:** Anschutz Medical Center U of Colorado Vivent Health Denver, Denver, Colorado; Washington University School of Medicine, Saint Louis, Missouri; Vivent Health, Des Moine, Iowa; Vivent Health, Des Moine, Iowa; Vivent Health, Des Moine, Iowa; Vivent Health, Des Moine, Iowa; Vivent Health, Des Moine, Iowa; Vivent Health, Des Moine, Iowa; Vivent Health, Des Moine, Iowa; Viventh Health, Kenosha, Wisconsin; Vivent health, St Louis, Missouri; Vivent Health, Des Moine, Iowa; Vivent Health. Medical College of WI, Milwaukee, Wisconsin

## Abstract

**Background:**

The use of FTC/TAF (Descovy) was approved for PrEP by the FDA or men who sex with men (MSM). People of color (POC) are disproportionately affected by HIV and less likely to use PrEP due to barriers to access. Telehealth approaches may reduce barriers and improve uptake of PrPE in the MSM population.

Study Participants vs historical controls

**Figure:**
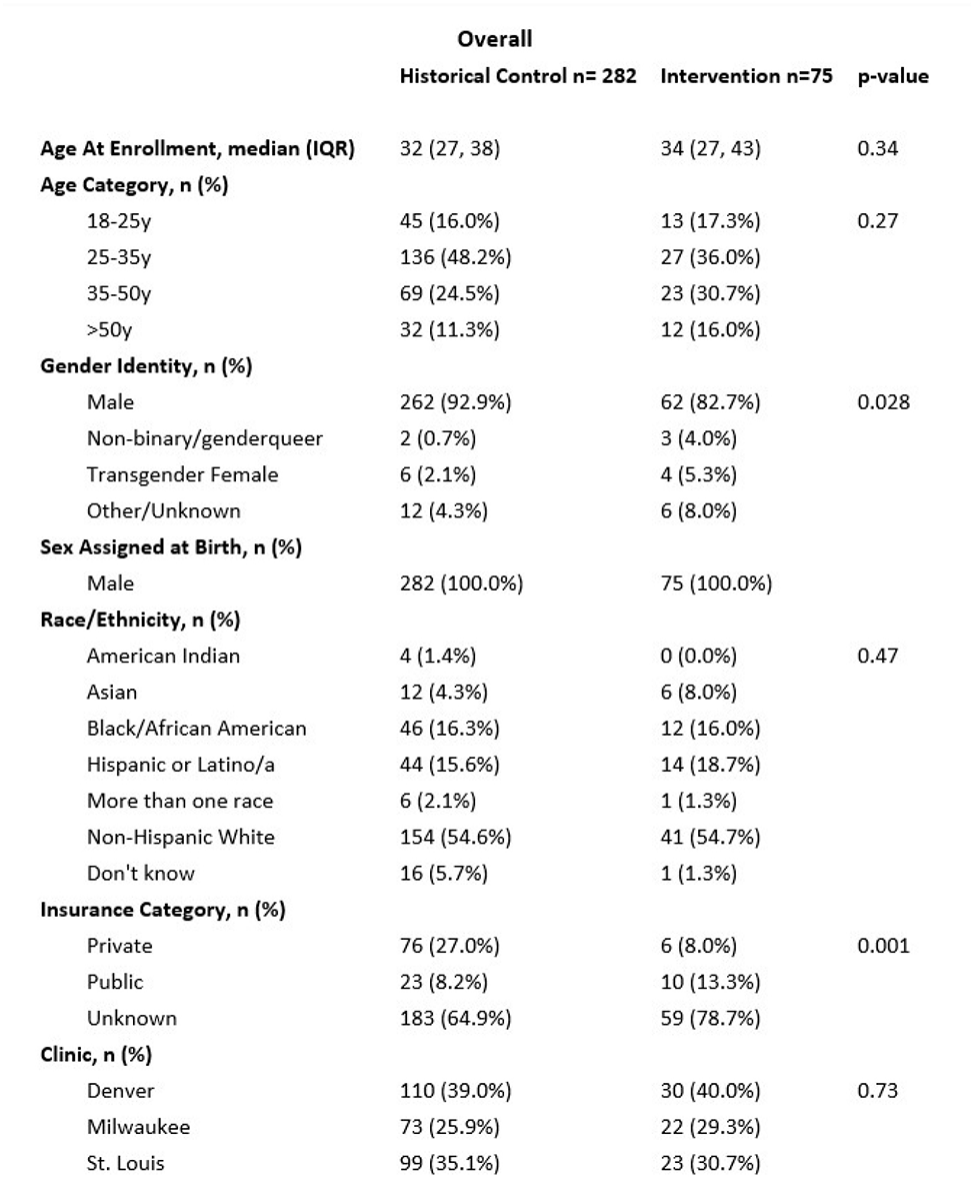

**Methods:**

We recruited MSM and transgender females who ere hIV negative, 18 years and older, had not used PrEP for > 30 days in the last year and had internet access from three Vivent Health clinics (Denver, Milwaukee and St Louis). We advertised the Telehealth PrEP intervention over social media, in-person at LGBTQIA+ events, and in surrounding rural areas. Participants were consented via the internet or in person. HIV testing was done vial oral swab, and PrEP was initiated during a Telehealth visit, and FTC/TAF was sent to their homes. A 3- month follow-up Telehealth visit was conducted. We describe demographics, retention at 3-months compared to historical controls who had initiated PrEP through routine care at these clinics using chi-square tests. An electronic Likert questionnaire was sent to study participants and staff.

**Results:**

Overall, 75 participants were recruited at three sites: 30 in Denver (40.0%), 22 in Milwaukee (29.3%) and 23 in St. Louis (30.7%). Median age was 34 years (IQR 27,43) and 62 were male (82.7%), 4 were transgender female (5.3%) and 3(4%) were nonbinary (male). Forty-one (54.7%) were non-Hispanic White, 12 (16.0%) were Black/African-American, 14(18.7%) were Latino and 6 (8.0%) were Asian. There were no HIV seroconversions among study participants: 48(64.0%) completed their 3-month follow-up visit (44 [93.6%] virtual only,1 [2.1%] in-person only, and 3 [6.3%] both). Demographics o those initiation PrEP were similar to historical controls. Almost 90% of study participants were satisfied with their telehealth visit and 100% would recommend Telehealth visit. While 100% of staff would recommend Telehealth, only 40% were satified with the telehealth visit.

**Conclusion:**

PrEP initiation and follow-up via Telehealth resulted in similar short-term persistence as routine in person care but was not more successful in improving prEP uptake among marginalized groups. Other modalities are needed to improve uptake of PrEP for POC.

**Disclosures:**

Cynthia Firnhaber, MD, MS, DTM&H, MERCK: Advisor/Consultant|MERCK: Grant/Research Support Aaloke Mody, MD, Gilead: Grant/Research Support

